# Plasmon damping depends on the chemical nature of the nanoparticle interface

**DOI:** 10.1126/sciadv.aav0704

**Published:** 2019-03-22

**Authors:** Benjamin Foerster, Vincent A. Spata, Emily A. Carter, Carsten Sönnichsen, Stephan Link

**Affiliations:** 1 Graduate School for Excellence Materials Science in Mainz, Johannes Gutenberg University Mainz, Staudinger Weg 9, D-55128 Mainz, Germany.; 2 Department of Mechanical and Aerospace Engineering, Princeton University, Princeton, NJ 08544-5263, USA.; 3 School of Engineering and Applied Science, Princeton University, Princeton, NJ 08544-5263, USA.; 4 Institute of Physical Chemistry, Johannes Gutenberg University Mainz, Duesbergweg 10-14, D-5128 Mainz, Germany.; 5 Department of Chemistry, Department of Electrical and Computer Engineering, Laboratory for Nanophotonics, Rice University, Houston, TX 77005, USA.

## Abstract

The chemical nature of surface adsorbates affects the localized surface plasmon resonance of metal nanoparticles. However, classical electromagnetic simulations are blind to this effect, whereas experiments are typically plagued by ensemble averaging that also includes size and shape variations. In this work, we are able to isolate the contribution of surface adsorbates to the plasmon resonance by carefully selecting adsorbate isomers, using single-particle spectroscopy to obtain homogeneous linewidths, and comparing experimental results to high-level quantum mechanical calculations based on embedded correlated wavefunction theory. Our approach allows us to indisputably show that nanoparticle plasmons are influenced by the chemical nature of the adsorbates 1,7-dicarbadodecaborane(12)-1-thiol (M1) and 1,7-dicarbadodecaborane(12)-9-thiol (M9). These surface adsorbates induce inside the metal electric dipoles that act as additional scattering centers for plasmon dephasing. In contrast, charge transfer from the plasmon to adsorbates—the most widely suggested mechanism to date—does not play a role here.

## Teaser

Damping of gold nanorod plasmons by surface-adsorbed molecules is best explained by scattering off adsorbate-induced dipoles.

## INTRODUCTION

Localized surface plasmons, the light-induced collective oscillation of conduction band electrons in metal nanoparticles, have had a tremendous impact on the field of chemistry: chemical-specific sensing became possible via surface-enhanced Raman scattering since its discovery in the 1970s (*1*–*5*), while surface plasmon–mediated hot carrier generation recently produced a subfield of photochemistry (*6*, *7*). Hot electrons and holes can selectively catalyze chemical reactions through charge or energy transfer to surface adsorbates by carefully matching plasmon resonance energies with molecular energy levels (*8*–*13*). Nevertheless, plasmons are described exclusively by electromagnetic theory that treats the surrounding chemical environment as an effective medium (*14*, *15*), neglecting any dependence on chemical composition. Although the influence of adsorbates on nanoparticle plasmons has been noted as far back as the 1980s (*15*–*17*), the even stronger size and shape dependence of plasmons coupled with ensemble measurements have thus far prevented a consistent, quantitative analysis and theoretical understanding.

A localized surface plasmon decays through radiation or by scattering with bulk defects and at the nanoparticle surface (*18*, *19*). The time scale of this dephasing is on the order of at most a few tens of femtoseconds (*18*), making direct time-resolved measurements difficult (*20*, *21*). An equivalent and more common approach to quantifying plasmon decay is to evaluate the resonance linewidth and intensity (*18*, *22*–*24*), as long as single-particle spectroscopy is used to yield nanoparticle intrinsic values free from ensemble averaging of heterogeneous size and shape distributions. When molecules chemically bind to the surfaces of metal nanoparticles, their plasmon decay can be accelerated as manifested by additional damping (i.e., increase in linewidth and decrease in intensity), referred to as chemical interface damping (*15*, *23*, *24*). However, plasmon damping by molecular adsorbates is still poorly understood and is often simply invoked when all other explanations fail. The obstacles hindering a quantitative understanding of chemical effects on plasmon damping are rooted in both experiment and theory. The latter requires a quantum mechanical approach for the electronic structure of molecules adsorbed on a metal surface, while the former suffers from the complexity that the different damping channels are highly interrelated (*18*, *19*, *25*, *26*). Specifically, bulk damping depends on the plasmon resonance energy, which, in turn, is affected by the refractive index of the surrounding medium (*25*). It is therefore difficult to isolate the difference in only chemical interface damping caused by different adsorbates.

## RESULTS AND DISCUSSION

**Fig. 1:**
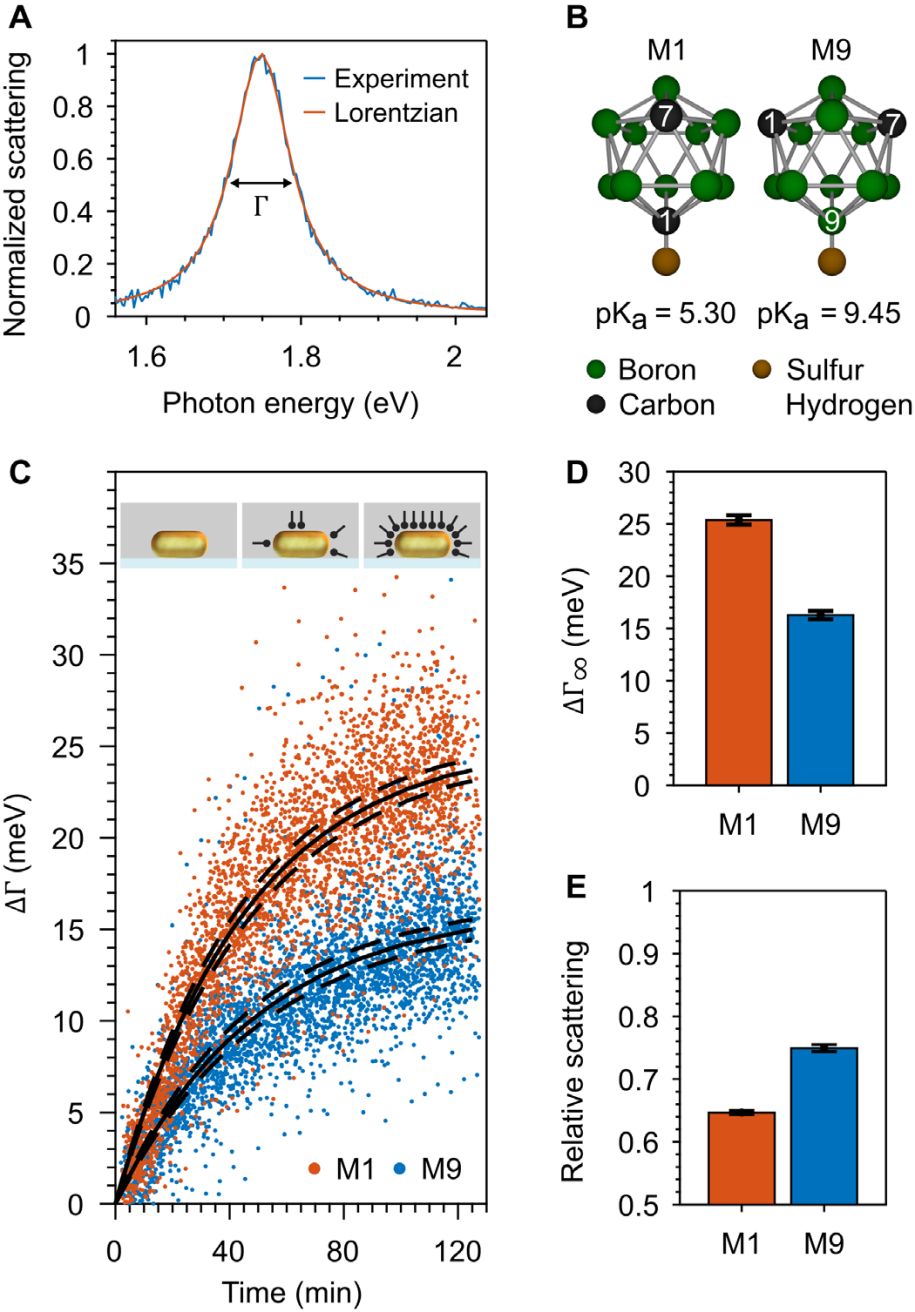
Plasmon energy loss through adsorbed M1 and M9 carboranethiols. (**A**) Representative normalized scattering spectrum of a 22 ± 2 nm by 66 ± 4 nm single gold nanorod. The plasmon linewidth Γ is determined from a fit to a Lorentzian function. (**B**) Ball-and-stick model of M1 and M9 carboranethiols with the hydrogen atoms omitted for clarity. The difference between the M1 and M9 isomers is the placement of the two carbon atoms in the boron cage. The thiol group (-SH) is more acidic when attached to a carbon atom (M1) than to a boron atom (M9). (**C**) Plasmon linewidth broadening over time ΔΓ(*t*) = Γ(*t*) − Γ(*t*_0_) of 115 and 130 single gold nanorods (red and blue dots) in M1 or M9 solutions (*t* > *t*_0_) compared to only solvent at the start of the measurement (*t*_0_). Langmuir adsorption isotherms (black lines) were used to estimate the adsorption time constant *k* and plasmon broadening ∆Γ_∞_ for a fully thiol-covered gold nanorod. Inset: Schematic illustration of an uncoated, partially, and fully carboranethiol-coated gold nanorod in a microfluidic cell. (**D**) Plasmon linewidth broadening ∆Γ_∞_ for fully M1 and M9 carboranethiol–covered gold nanorods obtained from the Langmuirian kinetics in (C). The dashed lines in (C) and error bars in (D) and (E) indicate the 95% confidence bounds of the fitted kinetics. The difference in plasmon broadening between M1 and M9 is clearly outside the error. (**E**) Mean scattering of 115 and 130 single gold nanorods after *t* = 114 to 120 min and *t* = 116 to 123 min in M1 and M9 solutions relative to the scattering intensity in ethanol at *t* = 0 min.

To overcome these challenges, we studied two chemical isomers with the same molecular size: 1,7-dicarbadodecaborane(12)-1-thiol and 1,7-dicarbadodecaborane(12)-9-thiol (M1 and M9 in Fig. 1B, respectively). These carboranethiols have the same size, geometry, and binding group, and scanning tunneling microscopy revealed that they adsorb with the same density on gold surfaces (*27*, *28*). M1 and M9 are, however, chemically different. Both carboranethiols consist of an icosahedral boron cage, in which two boron atoms are replaced by positively charged carbon atoms (*27*–*29*). The sulfur atom in M1 is attached to a carbon atom giving it more electronegative character and higher acidity (p*K*_a_ = 5.30) compared to M9 (p*K*_a_ = 9.45), in which the sulfur atom is attached to a boron atom instead (*29*). By comparing the change in homogeneous plasmon linewidth of single gold nanorods due to adsorption of these two carboranethiols, we are able to quantitatively determine chemical interface damping and interpret the mechanism based on calculations obtained with embedded correlated wavefunction (ECW) theory. In particular, we find that charge transfer, invoked as the main explanation of chemical interface damping in many instances before (*7*, *12*, *30*), does not play a role here. Instead, the plasmon is damped by scattering off dipoles induced by the carboranethiols.

Plasmon damping was determined from the linewidth Γ of single-particle dark-field scattering spectra (Fig. 1A). Because we measured the same individual gold nanorods (22 ± 2 nm by 66 ± 4 nm; section S1) after removal of the initial surfactants and during the entire carboranethiol adsorption process (see Methods), we only need to consider changes in linewidth ∆Γ and can equate them directly to plasmon damping by M1 and M9 while ignoring all other energy loss pathways. A small difference in resonance energy shift (fig. S4) can be neglected for plasmon energies below 1.76 eV (*31*). We observed the plasmon linewidth of 115 and 130 single gold nanorods for more than 120 min during M1 and M9 carboranethiol adsorption, respectively (Fig. 1C). The plasmon linewidth broadened over time *t*, ΔΓ(*t*), following kinetics typical for a Langmuir adsorption isotherm, i.e., ΔΓ(*t*) = ΔΓ_∞_[1 − exp(−*kt*)] (*23*). On the basis of Langmuirian kinetics, we obtained the plasmon broadening of fully carboranethiol-covered gold nanorods at infinite time ΔΓ_∞_ and the adsorption time constant *k*. An alternative way to visualize the difference in plasmon damping for the M1 and M9 carboranethiols is to plot the histograms of all single-particle linewidths measured before and after 120 min (section S3).

M1 carboranethiols damp plasmons 56% more strongly than M9 carboranethiols. We obtain values of ΔΓ_∞,M1_ = 25.4 ± 0.4 meV and ΔΓ_∞,M9_ = 16.3 ± 0.4 meV for gold nanorods covered with M1 or M9 carboranethiols (Fig. 1D). The relative scattering intensity was also reduced to 65 and 75% after *t* = 114 to 120 min and *t* = 116 to 123 min for the M1 and M9 carboranethiols, respectively (Fig. 1E) (*23*). The larger reduction in scattering intensity for M1-coated gold nanorods is in quantitative agreement with the stronger damping inferred from the increase in linewidth (*23*). Consistent changes in both linewidth and intensity as well as comparable resonance energy shifts (fig. S4) support the conclusion that the plasmon more strongly interacts with the more electronegative M1 carboranethiols.

**Fig. 2:**
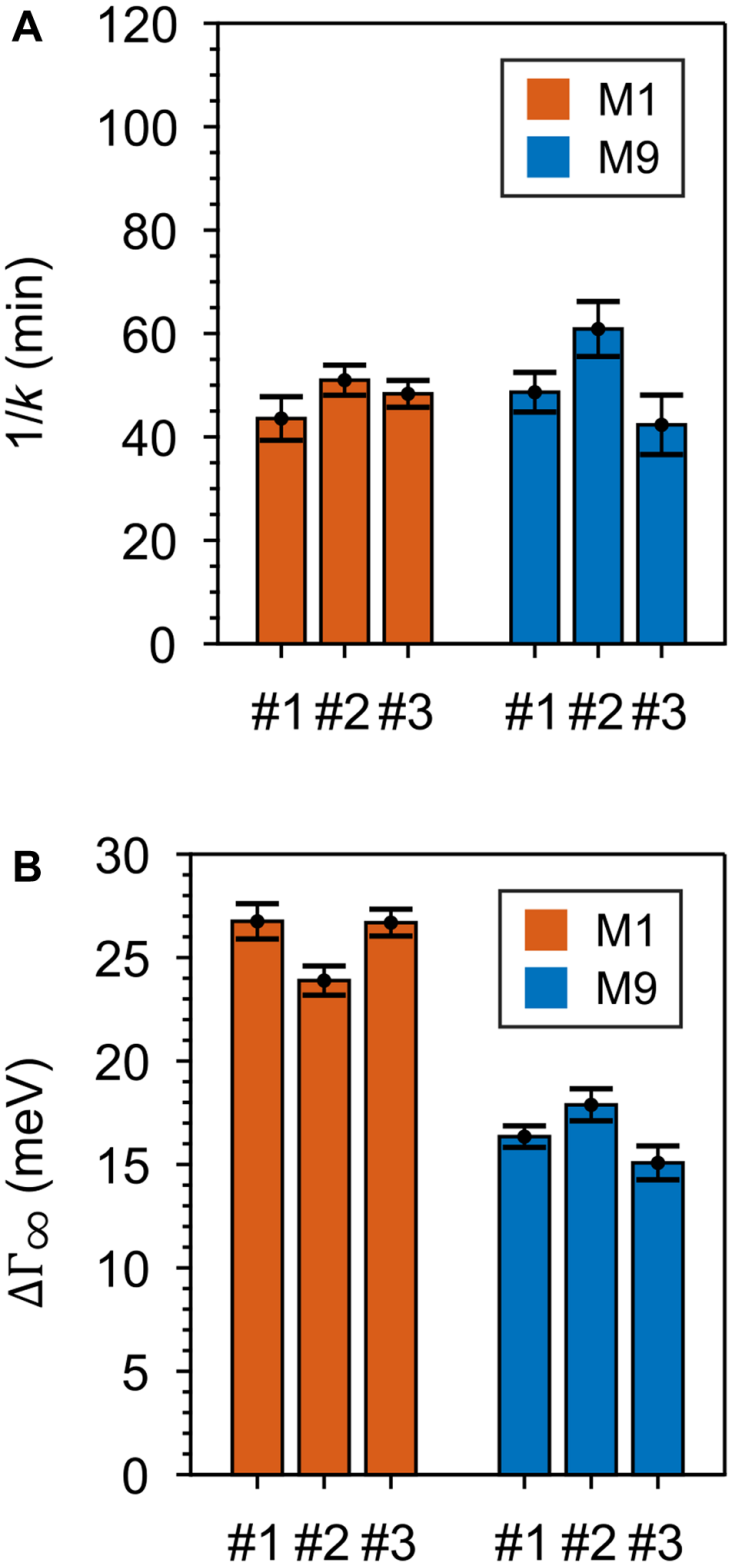
Complete coverage of carboranethiols on gold nanorods. (**A**) Adsorption time constant *k* from three independent adsorption experiments for M1 (red) and M9 (blue) carboranethiols (#1, #2, and #3). The adsorption time constants are similar in each experiment and reproducible, meaning that both carboranethiols adsorbed quickly and in similar fashion on the gold nanorods. (**B**) Plasmon broadening at infinite time ∆Γ_∞_ for fully M1 (red) and M9 (blue) carboranethiol–coated gold nanorods from three independent experiments (#1, #2, and #3) obtained from Langmuir adsorption kinetics. The plasmon linewidth broadening ∆Γ_∞_ differs between M1 and M9 carboranethiols but is consistent among the three experiments carried out for each carboranethiol. Corresponding adsorption isotherms with Langmuirian kinetics are given in section S3.

The adsorption kinetics of both carboranethiols are highly reproducible and the same within our experimental error, implying a comparable coverage of adsorbed molecules. Complementary analytical methods that determine the density of adsorbates on individual gold nanoparticles and are applicable to our experimental measurement scheme do not exist (*32*). We therefore used the adsorption kinetics of the plasmon broadening as an intrinsic control to exclude that differences in ΔΓ_∞_ were not caused by different numbers of adsorbed molecules. The adsorption time constants *k* for the M1 and M9 carboranethiols (1/*k*_M1_ = 46 ± 2 min and 1/*k*_M9_ = 50 ± 3 min) are the same within their errors (Fig. 1C). The time constants *k* were reproducible in six independent adsorption experiments for both carboranethiols (Fig. 2A), while, at the same time, plasmon broadening was always stronger for M1 than M9 (Fig. 2B). This conclusion that M1 and M9 similarly adsorb to the gold nanorods is consistent with previous scanning tunneling microscopy measurements (*27*, *28*), especially when considering studies that have found gold nanorod surfaces to be mostly flat (*33*) and molecular coverages to be comparable between gold nanorods and bulk surfaces (*34*).

**Fig. 3:**
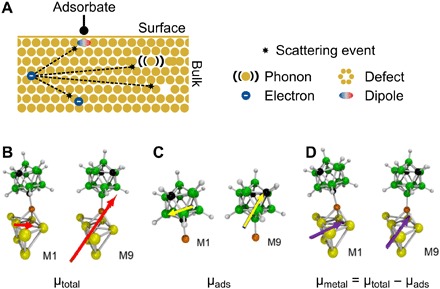
Magnitudes of induced surface dipoles in the metal determine plasmon damping of carboranethiols. (**A**) Schematic representation of plasmon damping in a nanorod: Conduction band electrons lose their energy by scattering with phonons, defects, and other electrons (*15*, *18*). Surface adsorbates create additional scattering centers in the form of induced dipoles (*35*). The scattering efficiency depends on the average distance an electron needs to travel to reach the induced dipoles, i.e., the nanorod surface (*15*, *18*, *23*, *51*). (**B** to **D**) ECW theory yields the total dipole moment μ_total_ of the M1 and M9 carboranethiols adsorbed on gold (B; red arrows) and in the gas phase (C; yellow arrows). The difference between these dipole moments gives the induced dipole moment in gold (D; violet arrows) due to carboranethiol adsorption (section S5).

Plasmons such as electrons scatter at defects (internal and at the particle surface), phonons, and electrons, losing their initial momentum and energy (Fig. 3A) (*15*, *18*). The frequency of these scattering events is described by the metal-specific electron mean free path and the effective particle size in the case of nanostructures. Nordlander and co-workers (*35*) proposed that electron-phonon relaxation in metal nanoparticles is enhanced when surface adsorbates induce electric dipoles at the nanoparticle surface. These dipoles act as additional scattering centers, and relaxation times were found to scale with the magnitude of the induced dipole moments. We therefore consider here the possibility that induced dipoles are created when the carboranethiols adsorb to the gold nanorods and similarly lead to accelerated plasmon decay.

Using ECW theory, we indeed find that dipoles are induced by the carboranethiols at the nanoparticle interface and that their magnitude and orientation differ for M1 and M9. Details of the calculations and the values of all electric dipoles are given in the sections S4 and S5, while Fig. 3 (B to D) summarizes the main result. Both carboranethiols have permanent electric dipole moments due to the substituted carbon atoms in the boron cage (*27*, *28*). When the carboranethiols adsorb on a metal surface, the freely moving conduction band electrons respond to the molecular dipole by creating image dipoles. The magnitude and orientation of these adsorbate-induced electric dipoles in the metal (Fig. 3D) are obtained by subtracting the electric dipoles of the carboranethiols in the gas phase (Fig. 3C) from the electric dipoles calculated for carboranethiols on an embedded gold cluster (Fig. 3B). It is justified to omit the ethanol solvent because it will interact only weakly with both the Au surface (ethanol only physisorbs on Au) and these inorganic boron carbides M1 and M9, resulting in a random orientation of solvent molecules with no net polarization.

Interactions between adsorbate-induced surface dipoles and the dipole of the experimentally probed longitudinal plasmon mode are maximized when they line up parallel to each other. To quantitatively assess the difference in plasmon damping for M1 and M9, we therefore consider the magnitude of the dipole component parallel to the long nanorod axis. We find that the induced electric dipoles along the gold surface plane μ*_xy_* are 4.03 and 2.74 D for M1 and M9 carboranethiols, respectively. The ratio of their magnitude μ_*xy*,M1_/μ_*xy*,M9_ = 1.47 is in quantitative agreement with the experimental observed plasmon broadening ratio ΔΓ_∞,M1_/ΔΓ_∞,M9_ = 1.56. We have considered here only the carboranethiols adsorbed at the nanorod sides because Zijlstra *et al.* (*24*) previously showed greatly reduced damping of end-only functionalized gold nanorods compared to functionalization at the sides and ends (see also section S1). It is also important to mention that these induced dipole moments, of course, do not directly predict the rate of electron scattering.

A long-standing theory by Persson (*17*), which links chemical interface damping with charge transfer into empty electronic states created via surface adsorption, agrees qualitatively with our results. However, this theory fails to quantify the difference in damping by M1 and M9 carboranethiols when we calculate, using density functional theory (DFT), the adsorbate density of states (see section S6). For the carboranethiols studied here, we therefore find no evidence for charge transfer to the adsorbate molecules following plasmon excitation. We cannot exclude though that a charge-transfer mechanism might dominate chemical interface damping in other systems.

We have shown by comparing the surface adsorption of two chemical isomers that, for metal nanoparticles, the nature of the chemical interface affects the loss of energy stored in a plasmon. Quantitative agreement was achieved with ECW theory calculations that correlate the degree of plasmon damping with the magnitude of the dipole moment induced in the metal parallel to the plasmon oscillation. The induced surface dipoles act as additional scattering centers for plasmon dephasing. Our results pave the way for a more detailed understanding of the chemical interface of plasmonic nanoparticles, necessary for the design and optimization of plasmon-driven chemistry.

## METHODS

### Materials

Sodium borohydride (NaBH_4_), hydrogen tetrachlorogold(III) (HAuCl_4_), 5-bromosalicylic acid, l-ascorbic acid, cetyltrimethylammonium bromide, m-Carborane-1-thiol (C_2_H_12_B_10_S), and m-Carborane-9-thiol (C_2_H_12_B_10_S) were purchased from Sigma-Aldrich. Two-hundred–proof ethanol was purchased from Thermo Fisher Scientific. Silver nitrate was purchased from Carl Roth. Ultrapure water produced by a Milli-Q Direct 8 system from Millipore was used in all experiments. All chemicals were used as received without further purification.

### Synthesis of gold nanorods

Gold nanorods were synthesized using the method by Ye *et al.* (*36*). The amounts of chemicals were used as given in the supporting information by Ye *et al.* (*36*) for the 22 nm by 66 nm gold nanorods also yielding 22 nm by 66 nm gold nanorods in our synthesis. Dimensions of gold nanorods were estimated by analyzing transmission electron microscope (FEI Tecnai G2 Spirit Twin) images using an image recognition tool of the MATLAB software. Details of the average dimensions of the synthesized gold nanorods are given in section S1.

### Single-particle spectroscopy

Scattering spectra of single gold nanorods were obtained using a custom-built dark-field microscope equipped with a spectrometer. Details of the custom-built microscope are given in section S2. A microfluidic cell was used in the experiments to change the liquid around the gold nanorods. Details regarding the fabrication of the microfluidic cells are given below. A low concentration solution of 22 nm by 66 nm gold nanorod was flushed through the microfluidic cell. Then, ethanol was flushed through the cell and gold nanorods attached to the glass substrate. Thirty-one to 46 gold nanorods were selected, and scattering spectra were obtained from each selected gold nanorod. These spectra were used as reference. Then, a 3 mM ethanolic solution of M1 or M9 carboranethiol was flushed through the microfluidic cell, and the spectra of the selected gold nanorods were repeatedly obtained for about 120 min. This experiment was repeated three times for both carboranethiols. The results of each separate experiment are given in section S3.

### Microfluidic cell

Two coverslips #1 (Menzel-Gläser) were connected with parafilm as a spacer. A CO_2_ laser cutter (Trotec Speedy 100) was used to cut channels in the parafilm and holes into the coverslips. The holes in the coverslip were placed at the end of each channel in the parafilm. The microfluidic cell was sealed by placing it on a hot plate at 120°C for a few seconds and applying pressure. Silicon tubings (NeoLab) were attached to the holes in the coverslip, and liquid was pulled through the cell using syringes.

### Theoretical methods

ECW theory was used for the calculations to obtain the dipole moments via density functional embedding theory (DFET) (*37*). The correlated wavefunction method used was the complete active space self-consistent field method (*38*). Starting structures for ECW calculations and calculations to obtain the density of states were obtained from periodic slab DFT using the Perdew-Burke-Ernzerhof exchange-correlation functional (*39*) with D3 dispersion corrections (*40*, *41*). Hartree-Fock theory (*42*) was used to obtain the gas-phase dipole moments. A comprehensive summary of the protocol is included in section S4. Vienna Ab Initio Simulation Package (*43*–*46*) was used for the periodic DFT and DFET optimization of the embedding potential using an in-house embedding potential optimization code (*47*). Molpro (*48*) was used for the ECW and gas-phase calculations. Additional software used includes VESTA (*49*) version 3.2.1 for visualizing densities and the calculated embedding potential, VMD (*50*) for plotting and rendering the dipoles, and MATLAB for data analysis.

## Supplementary Material

http://advances.sciencemag.org/cgi/content/full/5/3/eaav0704/DC1

Download PDF

## SUPPLEMENTARY MATERIALS

Supplementary material for this article is available at http://advances.sciencemag.org/cgi/content/full/5/3/eaav0704/DC1 Section S1. Dimensions of gold nanorods Section S2. Custom-built dark-field microscope Section S3. M1 and M9 carboranethiol adsorption experiments Section S4. Theoretical protocol Section S5. Dipole moments of carboranethiols and gold Section S6. Density of states and Persson theory Fig. S1. Dimensions of gold nanorods. Fig. S2. Representative TEM image of 22 ± 2 nm by 66 ± 4 nm chemically prepared gold nanorods. Fig. S3. Cumulative distributions of plasmon linewidths and resonance energies of all individual gold nanorods. Fig. S4. Plasmon resonance energy shift Δ*E*_res_ of gold nanorods during adsorption of M1 and M9 carboranethiols and during a control experiment in pure solvent (ethanol) without thiols. Fig. S5. Plasmon linewidth broadening ∆Γ of individual gold nanorods during adsorption of M1 and M9 carboranethiols (red and green circles) for all three M1 and M9 adsorption experiments. Fig. S6. Plasmon linewidth broadening ∆Γ of gold nanorods during adsorption of M1 and M9 carboranethiols and during a control experiment in pure solvent (ethanol) without thiols. Fig. S7. The projected DOS for the atoms comprising the M1 carboranethiol molecule adsorbed on Au in a face-centered cubic (fcc) hollow site, according to DFT-PBE-D3 calculations. Fig. S8. The projected DOS for the atoms comprising the M9 carboranethiol molecule adsorbed on Au in an fcc hollow site, according to DFT-PBE-D3. Fig. S9. Predicted projected DOS and Lorentzian functions for M1 and M9 molecules on Au(111). Table S1. Calculated dipole moment vector components in debye (D) for M1 and M9 carboranethiol molecules adsorbed on Au (“Total”) and in the gas-phase (“Gas-Phase Thiol”). References (*52*–*78*)

## References

[1] Jeanmaire D. L., Van Duyne R. P., Surface Raman spectroelectrochemistry. J. Electroanal. Chem. 84, 1–20 (1977).

[2] Kneipp K., Wang Y., Kneipp H., Perelman L. T., Itzkan I., Dasari R. R., Feld M. S., Single molecule detection using surface-enhanced Raman scattering (SERS). Phys. Rev. Lett. 78, 1667–1670 (1997).

[3] Nie S., Emory S. R., Probing single molecules and single nanoparticles by surface-enhanced Raman scattering. Science 275, 1102–1106 (1997). 9027306 10.1126/science.275.5303.1102

[4] Fleischmann M., Hendra P. J., McQuillan A. J., Raman spectra of pyridine adsorbed at a silver electrode. Chem. Phys. Lett. 26, 163–166 (1974).

[5] Jensen L., Aikens C. M., Schatz G. C., Electronic structure methods for studying surface-enhanced Raman scattering. Chem. Soc. Rev. 37, 1061–1073 (2008). 18443690 10.1039/b706023h

[6] Brongersma M. L., Halas N. J., Nordlander P., Plasmon-induced hot carrier science and technology. Nat. Nanotechnol. 10, 25–34 (2015). 25559968 10.1038/nnano.2014.311

[7] Kale M. J., Christopher P., Plasmons at the interface. Science 349, 587–588 (2015). 26250672 10.1126/science.aac8522

[8] Christopher P., Xin H., Linic S., Visible-light-enhanced catalytic oxidation reactions on plasmonic silver nanostructures. Nat. Chem. 3, 467–472 (2011). 21602862 10.1038/nchem.1032

[9] Mubeen S., Lee J., Singh N., Krämer S., Stucky G. D., Moskovits M., An autonomous photosynthetic device in which all charge carriers derive from surface plasmons. Nat. Nanotechnol. 8, 247–251 (2013). 23435280 10.1038/nnano.2013.18

[10] Marimuthu A., Zhang J., Linic S., Tuning selectivity in propylene epoxidation by plasmon mediated photo-switching of Cu oxidation state. Science 339, 1590–1593 (2013). 23539599 10.1126/science.1231631

[11] DuChene J. S., Tagliabue G., Welch A. J., Cheng W.-H., Atwater H. A., Hot hole collection and photoelectrochemical CO_2_ reduction with plasmonic Au/p-GaN photocathodes. Nano Lett. 18, 2545–2550 (2018). 29522350 10.1021/acs.nanolett.8b00241

[12] Wu K., Chen J., McBride J. R., Lian T., Efficient hot-electron transfer by a plasmon-induced interfacial charge-transfer transition. Science 349, 632–635 (2015). 26250682 10.1126/science.aac5443

[13] Tan S., Argondizzo A., Ren J., Liu L., Zhao J., Petek H., Plasmonic coupling at a metal/semiconductor interface. Nat. Photonics 11, 806–812 (2017).

[14] C. F. Bohren, D. R. Huffman, *Absorption and Scattering of Light by Small Particles* (Wiley-VCH Verlag GmbH & Co. KGaA, 1983).

[15] W. Kreibig, M. Vollmer, *Optical Properties of Metal Cluster* (Springer, Berlin, 1995).

[16] Charlé K.-P., Frank F., Schulze W., The optical properties of silver microcrystallites in dependence on size and the influence of the matrix environment. Ber. Bunsenges. Phys. Chem. 88, 350–354 (1984).

[17] Persson B. N. J., Polarizability of small spherical metal particles: Influence of the matrix environment. Surf. Sci. 281, 153–162 (1993).

[18] Hartland G. V., Optical studies of dynamics in noble metal nanostructures. Chem. Rev. 111, 3858–3887 (2011). 21434614 10.1021/cr1002547

[19] Hu M., Novo C., Funston A., Wang H., Staleva H., Zou S., Mulvaney P., Xia Y., Hartland G. V., Dark-field microscopy studies of single metal nanoparticles: Understanding the factors that influence the linewidth of the localized surface plasmon resonance. J. Mater. Chem. 18, 1949–1960 (2008). 18846243 10.1039/b714759g PMC2563424

[20] Sun Q., Yu H., Ueno K., Kubo A., Matsuo Y., Misawa H., Dissecting the few-femtosecond dephasing time of dipole and quadrupole modes in gold nanoparticles using polarized photoemission electron microscopy. ACS Nano 10, 3835–3842 (2016). 26878248 10.1021/acsnano.6b00715

[21] Aeschlimann M., Brixner T., Fischer A., Hensen M., Huber B., Kilbane D., Kramer C., Pfeiffer W., Piecuch M., Thielen P., Determination of local optical response functions of nanostructures with increasing complexity by using single and coupled Lorentzian oscillator models. Appl. Phys. B 122, 199 (2016).

[22] Crut A., Maioli P., del Fatti N., Vallée F., Optical absorption and scattering spectroscopies of single nano-objects. Chem. Soc. Rev. 43, 3921–3956 (2014). 24724158 10.1039/c3cs60367a

[23] Foerster B., Joplin A., Kaefer K., Celiksoy S., Link S., Sönnichsen C., Chemical interface damping depends on electrons reaching the surface. ACS Nano 11, 2886–2893 (2017). 28301133 10.1021/acsnano.6b08010

[24] Zijlstra P., Paulo P. M. R., Yu K., Xu Q.-H., Orrit M., Chemical interface damping in single gold nanorods and its near elimination by tip-specific functionalization. Angew. Chem. Int. Ed. 51, 8352–8355 (2012). 10.1002/anie.201202318 22777822

[25] Sönnichsen C., Franzl T., Wilk T., von Plessen G., Feldmann J., Wilson O., Mulvaney P., Drastic reduction of plasmon damping in gold nanorods. Phys. Rev. Lett. 88, 077402 (2002). 11863939 10.1103/PhysRevLett.88.077402

[26] Dondapati S. K., Ludemann M., Müller R., Schwieger S., Schwemer A., Händel B., Kwiatkowski D., Djiango M., Runge E., Klar T. A., Voltage-induced adsorbate damping of single gold nanorod plasmons in aqueous solution. Nano Lett. 12, 1247–1252 (2012). 22313237 10.1021/nl203673g

[27] Hohman J. N., Zhang P., Morin E. I., Han P., Kim M., Kurland A. R., McClanahan P. D., Balema V. P., Weiss P. S., Self-assembly of carboranethiol isomers on Au{111}: Intermolecular interactions determined by molecular dipole orientations. ACS Nano 3, 527–536 (2009). 19243128 10.1021/nn800673d

[28] Kim J., Rim Y. S., Liu Y., Serino A. C., Thomas J. C., Chen H., Yang Y., Weiss P. S., Interface control in organic electronics using mixed monolayers of carboranethiol isomers. Nano Lett. 14, 2946–2951 (2014). 24773449 10.1021/nl501081q

[29] Bregadze V. I., Dicarba-closo-dodecaboranes C_2_B_10_H_12_ and their derivatives. Chem. Rev. 92, 209–223 (1992).

[30] Hövel H., Fritz S., Hilger A., Kreibig U., Vollmer M., Width of cluster plasmon resonances: Bulk dielectric functions and chemical interface damping. Phys. Rev. B 48, 18178–18188 (1993). 10.1103/physrevb.48.18178 10008457

[31] Foerster B., Rutten J., Pham H., Link S., Sönnichsen C., Particle plasmons as dipole antennas: State Representation of Relative observables. J. Phys. Chem. C 122, 19116–19123 (2018).

[32] Richman E. K., Hutchison J. E., The nanomaterial characterization bottleneck. ACS Nano 3, 2441–2446 (2009). 19769400 10.1021/nn901112p

[33] Katz-Boon H., Rossouw C. J., Weyland M., Funston A. M., Mulvaney P., Etheridge J., Three-dimensional morphology and crystallography of gold nanorods. Nano Lett. 11, 273–278 (2011). 21182286 10.1021/nl103726k

[34] Djebaili T., Richardi J., Abel S., Marchi M., Atomistic simulations of the surface coverage of large gold nanocrystals. J. Phys. Chem. C 117, 17791–17800 (2013).

[35] Westcott S. L., Averitt R. D., Wolfgang J. A., Nordlander P., Halas N. J., Adsorbate-induced quenching of hot electrons in gold core-shell nanoparticles. J. Phys. Chem. B 105, 9913–9917 (2001).

[36] Ye X., Jin L., Caglayan H., Chen J., Xing G., Zheng C., Doan-Nguyen V., Kang Y., Engheta N., Kagan C. R., Murray C. B., Improved size-tunable synthesis of monodisperse gold nanorods through the use of aromatic additives. ACS Nano 6, 2804–2817 (2012). 22376005 10.1021/nn300315j

[37] Huang C., Pavone M., Carter E. A., Quantum mechanical embedding theory based on a unique embedding potential. J. Chem. Phys. 134, 154110 (2011). 21513378 10.1063/1.3577516

[38] Roos B. O., Taylor P. R., Si≐gbahn P. E. M., A complete active space SCF method (CASSCF) using a density matrix formulated super-CI approach. Chem. Phys. 48, 157–173 (1980).

[39] Perdew J. P., Burke K., Ernzerhof M., Generalized gradient approximation made simple. Phys. Rev. Lett. 77, 3865–3868 (1996). 10062328 10.1103/PhysRevLett.77.3865

[40] Grimme S., Antony J., Ehrlich S., Krieg H., A consistent and accurate ab initio parametrization of density functional dispersion correction (DFT-D) for the 94 elements H-Pu. J. Chem. Phys. 132, 154104 (2010). 20423165 10.1063/1.3382344

[41] Grimme S., Ehrlich S., Goerigk L., Effect of the damping function in dispersion corrected density functional theory. J. Comput. Chem. 32, 1456–1465 (2011). 21370243 10.1002/jcc.21759

[42] Roothaan C. C. J., New developments in molecular orbital theory. Rev. Mod. Phys. 23, 69–89 (1951).

[43] Kresse G., Furthmüller J., Efficiency of ab-initio total energy calculations for metals and semiconductors using a plane-wave basis set. Comput. Mater. Sci. 6, 15–50 (1996). 10.1103/physrevb.54.11169 9984901

[44] Kresse G., Furthmüller J., Efficient iterative schemes forab initiototal-energy calculations using a plane-wave basis set. Phys. Rev. B 54, 11169–11186 (1996). 10.1103/physrevb.54.11169 9984901

[45] Kresse G., Hafner J., Ab initiomolecular dynamics for liquid metals. Phys. Rev. B 47, 558–561 (1993). 10.1103/physrevb.47.558 10004490

[46] Kresse G., Hafner J., Ab initiomolecular-dynamics simulation of the liquid-metal-amorphous-semiconductor transition in germanium. Phys. Rev. B 49, 14251–14269 (1994). 10.1103/physrevb.49.14251 10010505

[47] Yu K., Libisch F., Carter E. A., Implementation of density functional embedding theory within the projector-augmented-wave method and applications to semiconductor defect states. J. Chem. Phys. 143, 102806 (2015). 26373999 10.1063/1.4922260

[48] Werner H.-J., Knowles P. J., Knizia G., Manby F. R., Schütz M., Molpro: A general-purpose quantum chemistry program package. WIREs Comput Mol Sci 2, 242–253 (2012).

[49] Momma K., Izumi F., *VESTA* 3for three-dimensional visualization of crystal, volumetric and morphology data. J. Appl. Crystallogr. 44, 1272–1276 (2011).

[50] Humphrey W., Dalke A., Schulten K., VMD: Visual molecular dynamics. J. Mol. Graph. 14, 33–38 (1996). 8744570 10.1016/0263-7855(96)00018-5

[51] Novo C., Gomez D., Perez-Juste J., Zhang Z., Petrova H., Reismann M., Mulvaney P., Hartland G. V., Contributions from radiation damping and surface scattering to the linewidth of the longitudinal plasmon band of gold nanorods: A single particle study. Phys. Chem. Chem. Phys. 8, 3540–3546 (2006). 16871343 10.1039/b604856k

[52] Coronado E. A., Schatz G. C., Surface plasmon broadening for arbitrary shape nanoparticles: A geometrical probability approach. J. Chem. Phys. 119, 3926–3934 (2003).

[53] Olson J., Dominguez-Medina S., Hoggard A., Wang L.-Y., Chang W.-S., Link S., Optical characterization of single plasmonic nanoparticles. Chem. Soc. Rev. 44, 40–57 (2015). 24979351 10.1039/c4cs00131a PMC4641313

[54] Rosman C., Prasad J., Neiser A., Henkel A., Edgar J., Sönnichsen C., Multiplexed plasmon sensor for rapid label-free analyte detection. Nano Lett. 13, 3243–3247 (2013). 23789876 10.1021/nl401354f

[55] Henkel A., Ye W., Khalavka Y., Neiser A., Lambertz C., Schmachtel S., Ahijado-Guzmán R., Sönnichsen C., Narrowing the plasmonic sensitivity distribution by considering the individual size of gold nanorods. J. Phys. Chem. C 122, 10133–10137 (2018).

[56] Link S., El-Sayed M. A., Spectral properties and relaxation dynamics of surface plasmon electronic oscillations in gold and silver nanodots and nanorods. J. Phys. Chem. B 103, 8410–8426 (1999).

[57] W. Demtroeder, Experimentalphysik 2. (2014).

[58] Blöchl P. E., Projector augmented-wave method. Phys. Rev. B 50, 17953–17979 (1994). 10.1103/physrevb.50.17953 9976227

[59] Methfessel M., Paxton A. T., High-precision sampling for Brillouin-zone integration in metals. Phys. Rev. B 40, 3616–3621 (1989). 10.1103/physrevb.40.3616 9992329

[60] Monkhorst H. J., Pack J. D., Special points for Brillouin-zone integrations. Phys. Rev. B 13, 5188–5192 (1976).

[61] Makov G., Payne M. C., Periodic boundary conditions inab initiocalculations. Phys. Rev. B 51, 4014–4022 (1995). 10.1103/physrevb.51.4014 9979237

[62] Neugebauer J., Scheffler M., Adsorbate-substrate and adsorbate-adsorbate interactions of Na and K adlayers on Al(111). Phys. Rev. B 46, 16067–16080 (1992). 10.1103/physrevb.46.16067 10003746

[63] Mete E., Yilmaz A., Danisman M. F., A van der Waals density functional investigation of carboranethiol self-assembled monolayers on Au(111). Phys. Chem. Chem. Phys. 18, 12920–12927 (2016). 27108565 10.1039/c6cp01485b

[64] N. W. Ashcroft, D. N. Mermin, *Solid State Physics* (Saunders College Publishing, Orlando, Florida, 1976).

[65] H.-J. Werner et al., MOLPRO, version 2015.1, a package of ab initio programs. (2015).

[66] C. M. Krauter, E. A. Carter; https://github.com/EACcodes/EmbeddingIntegralGenerator.

[67] Dunning Jr. T. H., Gaussian basis sets for use in correlated molecular calculations. I. The atoms boron through neon and hydrogen. J. Chem. Phys. 90, 1007–1023 (1989).

[68] Bergner A., Dolg M., Küchle W., Stoll H., Preuß H., Ab initio energy-adjusted pseudopotentials for elements of groups 13–17. Mol. Phys. 80, 1431–1441 (2006).

[69] Feller D., The role of databases in support of computational chemistry calculations. J. Comput. Chem. 17, 1571–1586 (1996).

[70] Schuchardt K. L., Didier B. T., Elsethagen T., Sun L., Gurumoorthi V., Chase J., Li J., Windus T. L., Basis set exchange: A community database for computational sciences. J. Chem. Inf. Model. 47, 1045–1052 (2007). 17428029 10.1021/ci600510j

[71] Figgen D., Rauhut G., Dolg M., Stoll H., Energy-consistent pseudopotentials for group 11 and 12 atoms: Adjustment to multi-configuration Dirac–Hartree–Fock data. Chem. Phys. 311, 227–244 (2005).

[72] Peterson K. A., Puzzarini C., Systematically convergent basis sets for transition metals. II. Pseudopotential-based correlation consistent basis sets for the group 11 (Cu, Ag, Au) and 12 (Zn, Cd, Hg) elements. Theor. Chem. Accounts 114, 283–296 (2005).

[73] Weigend F., Ahlrichs R., Balanced basis sets of split valence, triple zeta valence and quadruple zeta valence quality for H to Rn: Design and assessment of accuracy. Phys. Chem. Chem. Phys. 7, 3297–3305 (2005). 16240044 10.1039/b508541a

[74] Becke A. D., Density-functional thermochemistry. III. The role of exact exchange. J. Chem. Phys. 98, 5648–5652 (1993).

[75] Stephens P. J., Devlin F. J., Chabalowski C. F., Frisch M. J., Ab initio calculation of vibrational absorption and circular dichroism spectra using density functional force fields. J. Phys. Chem. 98, 11623–11627 (1994).

[76] J. D. Jackson, *Classical Electrodynamics* (Wiley, New York, ed. 3, 1998).

[77] Persson B. N. J., Schumacher D., Otto A., Surface resistivity and vibrational damping in adsorbed layers. Chem. Phys. Lett. 178, 204–212 (1991). 10.1103/physrevb.44.3277 9999927

[78] D. R. Lide, *CRC Handbook of Chemistry and Physics* (CRC, Boca Raton, FL, 1995).

