# Supplementary material for: Plasmon damping depends on the chemical nature of the nanoparticle interface
Source: Sci Adv. 2019 Mar 22;5(3):eaav0704. doi: 10.1126/sciadv.aav0704 (PMC6430627; doi:10.1126/sciadv.aav0704)
Supplement: http://advances.sciencemag.org/cgi/content/full/5/3/eaav0704/DC1 [file supp_5_3_eaav0704__index.html]

Science Advances | Science Advances

## Supplementary Materials

**This PDF file includes:**

- Section S1. Dimensions of gold nanorods
- Section S2. Custom-built dark-field microscope
- Section S3. M1 and M9 carboranethiol adsorption experiments
- Section S4. Theoretical protocol
- Section S5. Dipole moments of carboranethiols and gold
- Section S6. Density of states and Persson theory
- Fig. S1. Dimensions of gold nanorods.
- Fig. S2. Representative TEM image of 22 ± 2 nm by 66 ± 4 nm chemically prepared gold nanorods.
- Fig. S3. Cumulative distributions of plasmon linewidths and resonance energies of all individual gold nanorods.
- Fig. S4. Plasmon resonance energy shift Δ*E*res of gold nanorods during adsorption of M1 and M9 carboranethiols and during a control experiment in pure solvent (ethanol) without thiols.
- Fig. S5. Plasmon linewidth broadening ∆Γ of individual gold nanorods during adsorption of M1 and M9 carboranethiols (red and green circles) for all three M1 and M9 adsorption experiments.
- Fig. S6. Plasmon linewidth broadening ∆Γ of gold nanorods during adsorption of M1 and M9 carboranethiols and during a control experiment in pure solvent (ethanol) without thiols.
- Fig. S7. The projected DOS for the atoms comprising the M1 carboranethiol molecule adsorbed on Au in a face-centered cubic (fcc) hollow site, according to DFT-PBE-D3 calculations.
- Fig. S8. The projected DOS for the atoms comprising the M9 carboranethiol molecule adsorbed on Au in an fcc hollow site, according to DFT-PBE-D3.
- Fig. S9. Predicted projected DOS and Lorentzian functions for M1 and M9 molecules on Au(111).
- Table S1. Calculated dipole moment vector components in debye (D) for M1 and M9 carboranethiol molecules adsorbed on Au (“Total”) and in the gas-phase (“Gas-Phase Thiol”).
- References (*52*–*78*)

Download PDF

**Files in this Data Supplement:**

- Adobe PDF - aav0704\_SM.pdf
