# Supplementary material for: Plasmon damping depends on the chemical nature of the nanoparticle interface
Source: Sci Adv. 2019 Mar 22;5(3):eaav0704. doi: 10.1126/sciadv.aav0704 (PMC6430627; doi:10.1126/sciadv.aav0704)
Supplement: Download PDF [file aav0704_SM.pdf]

Science Advances
AAAS

advances.sciencemag.org/cgi/content/full/5/3/eaav0704/DC1

Supplementary Materials for

Plasmon damping depends on the chemical nature of the nanoparticle interface

Benjamin Foerster, Vincent A. Spata, Emily A. Carter*, Carsten Sönnichsen*, Stephan Link*

*Corresponding author. Email: eac@princeton.edu (E.A.C.); soennichsen@uni-mainz.de (C.S.); slink@rice.edu (S.L.)

Published 22 March 2019, *Sci. Adv.* **5**, eaav0704 (2019)
DOI: 10.1126/sciadv.aav0704

This PDF file includes:

Section S1. Dimensions of gold nanorods
Section S2. Custom-built dark-field microscope
Section S3. M1 and M9 carboranethiol adsorption experiments
Section S4. Theoretical protocol
Section S5. Dipole moments of carboranethiols and gold
Section S6. Density of states and Persson theory
Fig. S1. Dimensions of gold nanorods.
Fig. S2. Representative TEM image of 22 ± 2 nm by 66 ± 4 nm chemically prepared gold nanorods.
Fig. S3. Cumulative distributions of plasmon linewidths and resonance energies of all individual gold nanorods.
Fig. S4. Plasmon resonance energy shift ΔE_{res} of gold nanorods during adsorption of M1 and M9 carboranethiols and during a control experiment in pure solvent (ethanol) without thiols.
Fig. S5. Plasmon linewidth broadening $\Delta\Gamma$ of individual gold nanorods during adsorption of M1 and M9 carboranethiols (red and green circles) for all three M1 and M9 adsorption experiments.
Fig. S6. Plasmon linewidth broadening $\Delta\Gamma$ of gold nanorods during adsorption of M1 and M9 carboranethiols and during a control experiment in pure solvent (ethanol) without thiols.
Fig. S7. The projected DOS for the atoms comprising the M1 carboranethiol molecule adsorbed on Au in a face-centered cubic (fcc) hollow site, according to DFT-PBE-D3 calculations.
Fig. S8. The projected DOS for the atoms comprising the M9 carboranethiol molecule adsorbed on Au in an fcc hollow site, according to DFT-PBE-D3.
Fig. S9. Predicted projected DOS and Lorentzian functions for M1 and M9 molecules on Au(111).
Table S1. Calculated dipole moment vector components in debye (D) for M1 and M9 carboranethiol molecules adsorbed on Au ("Total") and in the gas-phase ("Gas-Phase Thiol").
References (*52–78*)

Section S1. Dimensions of gold nanorods

PHYSICAL DIMENSIONS

Gold nanorods with an average size of 22±2 by 66±4 nm and an average aspect ratio of 3.1±0.3 were used in the experiments. The average dimensions of the chemically synthesized gold nanorods were determined from transmission electron microscopy (TEM) images. The TEM images were analyzed with an image recognition tool from the software MATLAB to determine the diameter, length, and aspect ratio of each gold nanorod in the TEM image. The histograms in fig. S1 show the diameter, length, and aspect ratio of 484 individual gold nanorods. A Gaussian distribution was used to determine an average diameter of 22±2 nm, average length of 66±4 nm, and average aspect ratio of 3.1±0.3. The size distribution of the gold nanorods used in this work is comparable to the one reported for the original synthesis from Murray and coworkers (diameter of 22±1 nm and length of 64±5 nm) (*36*). A representative TEM image used in the analysis is shown in fig. S2.

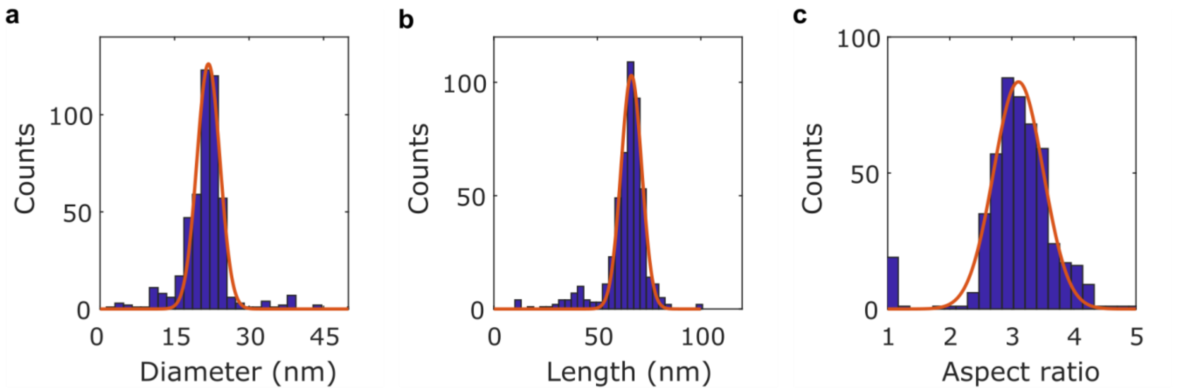

Fig. S1. Dimensions of gold nanorods. Histograms of the diameter (a), length (b), and aspect ratio (c) of 484 gold nanorods determined by TEM. A Gaussian distribution (red line) was used to determine the average values and standard deviations.

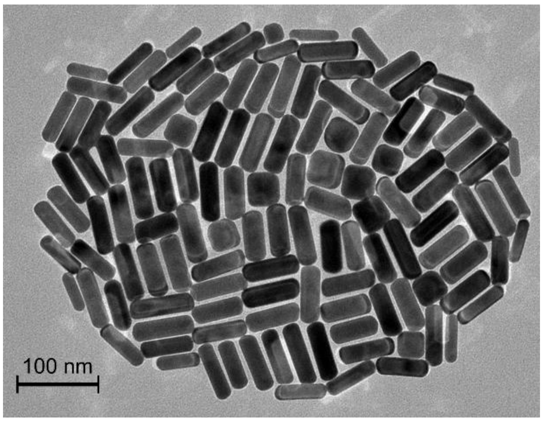

Fig. S2. Representative TEM image of 22 ± 2 nm by 66 ± 4 nm chemically prepared gold nanorods.

EFFECTIVE PATH LENGTH OF GOLD NANORODS

The effective path length of electrons to the surface l_{eff} describes the average distance an electron needs to travel before reaching the surface of the particle. The probability of an electron reaching the particle surface is proportional to the inverse of the effective path length (*15, 18, 19*). The inverse of the effective path length is proportional to the surface-to-volume ratio for an arbitrarily shaped particle and is given by the following expression (*52*)

$$1/l_{eff} = S/4V \qquad \text{(S1)}$$

If a gold nanorod is approximated as a cylinder, then the surface area S and volume V can expressed in terms of the nanorod width d and aspect ratio AR so that the effective path length reduces to

$$1/l_{eff} = 1/d + 1/(2 \cdot d \cdot AR) \qquad \text{(S2)}$$

When the aspect ratio of the gold nanorod becomes infinite, the probability of an electron reaching the surface only depends on the diameter, because the second term in Eq. (S2) becomes zero. In other words, the first term describes the probability of an electron reaching the side of the gold nanorod, while the second term describes the probability of an electron reaching the tips of the gold nanorod. In our case of gold nanorods with a diameter of 22 nm and an aspect ratio of 3.1, about 86 % of the electrons reach the sides and 14 % of the electrons reach the tips. Although counterintuitive considering the larger electric fields at the nanorod ends, surface damping is, however, more affected by adsorbates positioned at the sides.

Section S2. Custom-built dark-field microscope

Dark field contrast was used to collect scattered light of single gold nanorods. A dark field microscope (Zeiss Axio Observer Z1) was equipped with a digital single-lens reflex (DSLR) camera and a spectrometer. The principles of single-particle scattering spectroscopy are given in excellent reviews by Olson *et al*. and Lien-Crut *et al*. (*22, 53*). Here, we give a short technical description of our custom-built microscope, which has been described previously (*23, 54, 55*). A halogen lamp was used for white light illumination. The white light passed through a dark field condenser. This condenser created a hollow cone of light, which was focused on the gold nanorods dispersed on the bottom slide of a microfluidic cell. After the focal plane, the white light diverged into a hollow cone again and passed the oil-immersion objective (Zeiss Plan-Apochromat 40x/1.3), creating the dark-field contrast. However, the scattered light of the gold nanorods was collected by the oil-immersion objective. The scattered light of single gold nanorods was diffraction-limited, and therefore required that the particles needed to be sufficiently separated (roughly about 2 µm) to collect the scattered light from a single gold nanorod. The collected scattered light could be either directed to a DSLR camera (Canon EOS 5D Mark II) or a charge-coupled device (CCD) camera following a grating (Andor Luca R EM-CCD) to take a real color image of the sample or a spectrum of a single gold nanorod, respectively.

MATLAB software was used to automatically take spectra of single gold nanorods. The software identified the scattered light of gold nanorods as bright spots in the real color image. A PI542 XY-Piezo stage (Physik Instrumente, 200 µm x 200 µm travel range) moved the sample to the position of the first bright spot and automatically focused via a PI721 Z-Piezo objective driver (Physik Instrumente, 100 µm travel range). Then the scattered light of the gold nanorods passed through a slit and was spectrally resolved and collected by the CCD camera, while the scattered light from other parts of the sample was blocked by the slit. Once the single-particle spectra were successfully taken, the software automatically moved to the next particle. After the scattering spectra of all selected gold nanorods were taken (one run), the next run could be started with a delay (e.g., for a quick analysis of the data) or without a delay. The latter mode was mostly used during the experiments to increase the time resolution (time between taking two spectra of the same gold nanorod).

The number of gold nanorods measured for each experiment was carefully chosen to achieve good statistical sampling and time resolution. The time resolution depends on moving to a particle and bringing it in focus, the exposure time for taking a spectrum, and the number of gold nanorods measured per run. While the exposure time mostly depends on the scattering efficiency of the gold nanorods and should be long enough to obtain spectra with a good signal-to-noise ratio, the number of gold nanorods acquired per experiment was changed. We chose to measure 31 to 46 gold nanorods per experiment to account for a balance between time resolution (216-306 seconds under these conditions) and statistics. The adsorption experiment with M1 and M9 carboranethiols were repeated three times to further increase statistical sampling and to make sure that the results were reproducible (Supplementary Materials S3).

Section S3. M1 and M9 carboranethiol adsorption experiments

CUMULATIVE DISTRIBUTIONS OF RESONANCE ENERGY AND PLASMON LINEWIDTH

The plasmon linewidth and resonance energy of 115 and 130 gold nanorods were observed during adsorption of M1 and M9 carboranethiols. The average plasmon linewidth Γ was 89±6 meV and 87±6 meV at the beginning of the experiment and 112±6 meV and 102±6 meV at the end of the experiment after adsorption of M1 or M9 carboranethiol, respectively. The average resonance energy was 1.72±0.06 eV and 1.73±0.05 eV. This narrow distribution of both spectroscopic observables (fig. S3) is caused by the narrow size distribution and high crystallinity of the gold nanorod sample used in these experiments (Supplementary Materials S1).

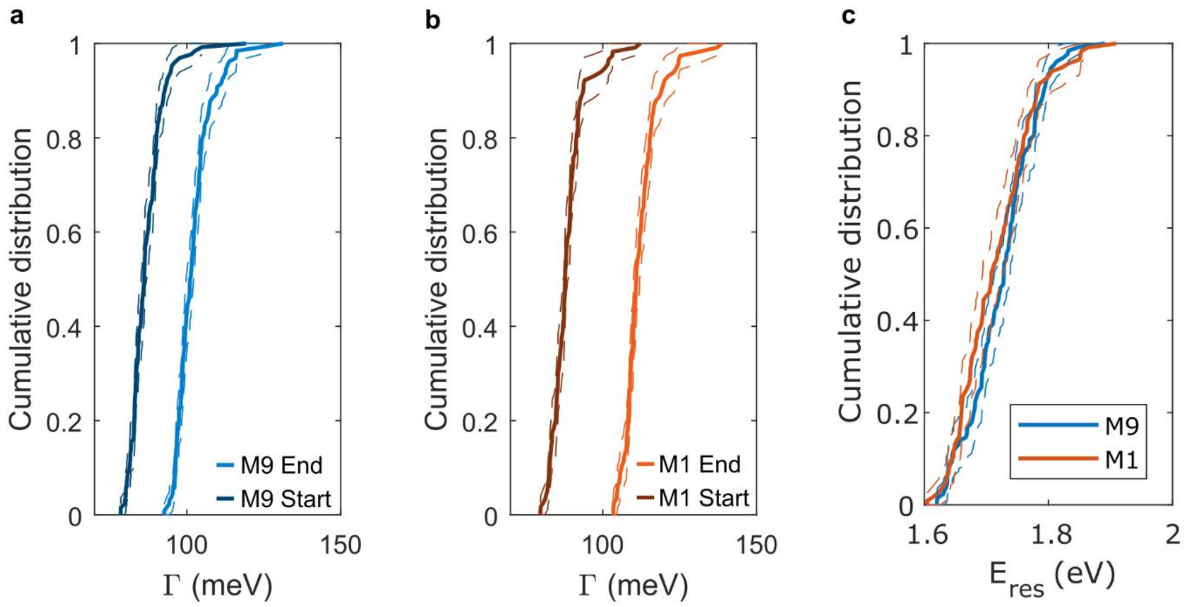

Fig. S3. Cumulative distributions of plasmon linewidths and resonance energies of all individual gold nanorods. a) Cumulative distribution of plasmon linewidths before adsorption of M9 carboranethiol (dark blue) and at the end of the experiment (light blue) after *t*=116-123 minutes. The dotted lines indicate the 95% confidence bounds. The average plasmon linewidth was 87±6 meV at the beginning and 102±6 meV at the end of the experiment. b) Cumulative distribution of plasmon linewidths before adsorption of M1 carboranethiol (dark red) and at the end of the experiment (light red) after *t*=114-120 minutes. The dotted lines indicate the 95% confidence bounds. The average plasmon linewidth was 89±6 meV at the beginning and 112±6 meV at the end of the experiment. The difference in plasmon damping between M1 and M9 as calculated simply from the change between the start and the end of the experiment is consistent with the value in the text obtained from fitting the adsorption kinetics (Fig. 1). c) Cumulative distribution of resonance energies at the beginning of the experiments before carboranethiol adsorption. The average resonance energy was 1.72±0.06 and 1.73±0.05 eV for M1 and M9 carboranethiol, respectively.

RESONANCE ENERGY SHIFT DURING ADSORPTION

The backbone of the carboranethiol changed the local refractive index around the gold nanorods, which led to a shift of the plasmon resonance to lower energies. M1 and M9 carboranethiols have a higher refractive index than ethanol. When the carboranethiols adsorbed on the gold nanorod surface in a dense layer, a coating with a higher refractive index was created. It is well known that an increase in refractive index leads to a red-shift of the plasmon resonance (*23*, *24*, *54*, *56*). The more carboranethiol molecules adsorbed on the gold nanorod surface over time *t*, the larger the plasmon resonance shift ΔE_{res} toward lower energies (fig. S4). This red-shift was observed for all three

experiments of both carboranethiols (M1 and M9) and was consistent between each experiment. It appeared that the M1 carboranethiol slightly red-shifted the plasmon resonance more than the M9 carboranethiol, although the errors of the resonance energy shifts for M1 and M9 overlap. This small redshift is in quantitative agreement with a slightly larger refractive index of M1 as determined from dilute ethanol solutions of M1 and M9 (not shown). The difference in resonance energies of 4 meV has no effect on the plasmon linewidth as we selected nanorods with plasmon energies smaller than 1.76 eV. We have shown previously that the linewidth becomes independent of resonance energy if far enough removed from interband transitions (*23, 31*). It is also possible that the damping of the plasmon resonance itself could lead to the observed small difference in red-shifts. It is known from dipolar resonances that damping leads to a small red-shift (*23, 57*). Because M1 damps the plasmon resonance more than M9, this increased damping could cause a larger red-shift.

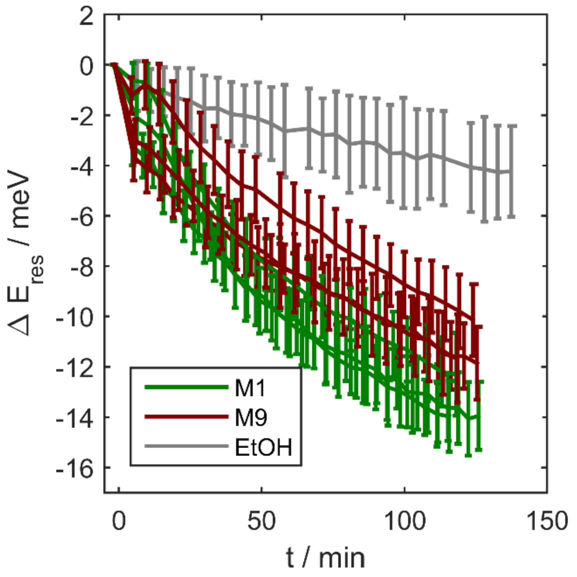

Fig. S4. Plasmon resonance energy shift ΔE_{res} of gold nanorods during adsorption of M1 and M9 carboranethiols and during a control experiment in pure solvent (ethanol) without thiols. Three experiments were conducted for each carboranethiol and between 31 and 46 gold nanorods were consecutively measured in each experiment. Once all gold nanorods were measured for one time (one run), all gold nanorods were measured again. The plasmon resonance energy shift compared to the first run was first determined for every single gold nanorod individually according to $\Delta E_{res} = E_{res}(t) - E_{res}(t_0)$ and then averaged over all single nanorods for each run. Error bars indicate the standard deviation.

Pure ethanol slightly red-shifts the plasmon resonance over time. Gold nanorods were measured in a control experiment in pure ethanol. The average plasmon resonance of these gold nanorods slightly shifted toward lower energies over time as well (fig. S4). This red-shift was significantly smaller than for the carboranethiols, but nevertheless unexpected. We hypothesize that ethanol could also physisorb onto the gold nanorod surface. This adsorption caused minor plasmon damping (fig. S6), therefore leading to a small red-shift of the plasmon resonance. A red-shift due to a refractive index change was unlikely because ethanol as the solvent was present at all times during the control experiments.

PLASMON LINEWIDTH SHIFT DURING ADSORPTION

Adsorption experiments were reproducible for each carboranethiol. M1 and M9 carboranethiols were adsorbed on gold nanorods and the plasmon broadening $\Delta\Gamma$ was observed over time t. These

experiments were repeated for both carboranethiols three times and are shown in fig. S5. The adsorption process (observed by the plasmon broadening ΔΓ) was consistent between all three experiments and the larger plasmon broadening by the M1 carboranethiol was clearly distinguishable from the plasmon broadening by the M9 carboranethiol. For further quantification, the adsorption curves were fitted with a Langmuir adsorption isotherm to extract the adsorption time constant *k* and equilibrium plasmon broadening $\Delta\Gamma_{CID}$. We found the adsorption time constant *k* and equilibrium broadening $\Delta\Gamma_{CID}$ to be very similar between experiments (Fig. 2; please see main text for details). Although the size distribution of gold nanorods is narrow (Supplementary Materials S1), the nevertheless existing heterogeneity of the gold nanorod sample most likely caused the small deviations of plasmon broadening between experiments.

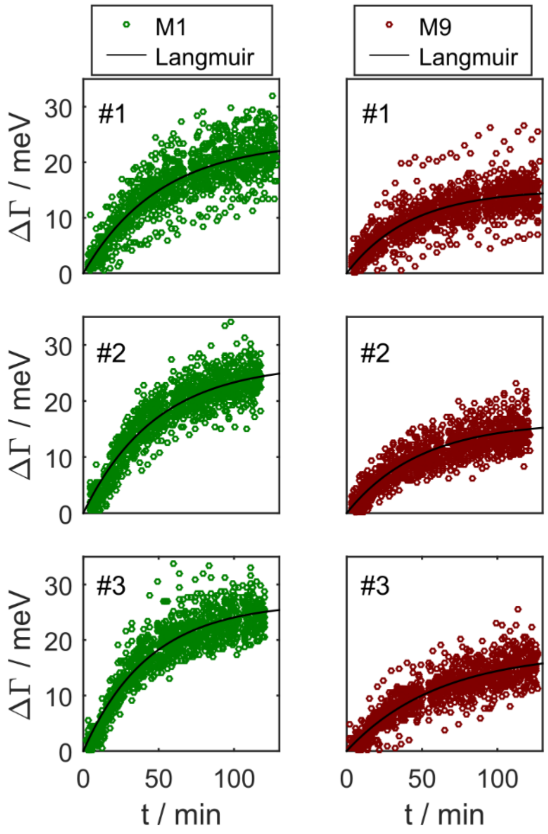

Fig. S5. Plasmon linewidth broadening ΔΓ of individual gold nanorods during adsorption of M1 and M9 carboranethiols (red and green circles) for all three M1 and M9 adsorption experiments. The combined data from these experiments is shown in Fig. 2. The adsorption curves were fitted with a Langmuir adsorption isotherm (black line) to extract the adsorption time constant *k* (M1 #1-3: 51±4 min, 48±3 min, 44±3 min; M9 #1-3: 42±4 min, 49±5 min, 61±6 min) and equilibrium plasmon broadening $\Delta\Gamma_{CID}$ (M1 #1-3: 23.9±0.9 meV, 26.7±0.7 meV, 26.8±0.6 meV; M9 #1-3: 15.1±0.5 meV, 16.3±0.8 meV, 17.9±0.8 meV).

The plasmon resonance broadens in the control experiment in pure ethanol but the effect is smaller compared to the adsorption of carboranethiols. Gold nanorods were measured in a control experiment in pure ethanol over time. The plasmon resonance broadened over time (fig. S6). This behavior is unexpected. We hypothesize that ethanol physisorbs on the gold nanorod surface and damps the plasmon resonance, leading to plasmon resonance broadening ΔΓ. Importantly though, the plasmon broadening by ethanol was significantly smaller than the damping caused by adsorption of carboranthiols, allowing us to confidently measure the difference between the M1 and M9 carboranethiols.

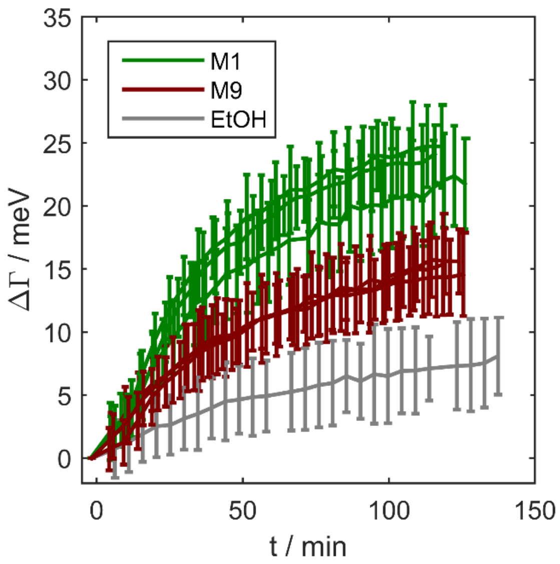

Fig. S6. Plasmon linewidth broadening ΔΓ of gold nanorods during adsorption of M1 and M9 carboranethiols and during a control experiment in pure solvent (ethanol) without thiols. Three experiments were conducted for each carboranethiol and between 31 and 46 gold nanorods were consecutively measured in each experiment. Once all gold nanorods were measured for one time (one run), the same gold nanorods were measured again. The plasmon linewidth broadening ΔΓ compared to the first run was first determined for every single gold nanorod individually according to $\Delta\Gamma=\Gamma(t) - \Gamma(t_0)$ and then averaged over all single gold nanorods for each run. Error bars indicate the standard deviation.

Section S4. Theoretical protocol

The Vienna Ab Initio Simulation Package (VASP) (*43–46*) version 5.3.5 was utilized to perform all-electron, frozen-core, projector-augmented-wave density functional theory (PAW-DFT) calculations (*58*), using the Perdew–Burke-Ernzerhof (PBE) exchange-correlation (XC) functional (*39*) and Grimme's semiempirical D3 dispersion corrections with Becke-Johnson damping vdW corrections (DFT+D3) (*40, 41*). Standard VASP PAW potentials were used that explicitly treat the 1*s* electron of hydrogen (H), 2*s* and 2*p* electrons of carbon (C) and boron (B), 3*s* and 3*p* electrons of sulfur (S), and 6*s* and 5*d* electrons of gold (Au). We used a kinetic energy cutoff of 600 eV for the planewave (PW) basis, with Methfessel-Paxton (*59*) Fermi surface smearing (width of 0.09 eV) and Brillouin zone sampling using the Monkhorst-Pack (*60*) method on a Γ-point-centered 7x7x1 k-mesh. These parameters converged total energies to within 1 meV/atom. The atomic forces for optimized structures were converged to within 0.01 eV/Å. Self-consistent dipole corrections (*61, 62*) were utilized to screen the dipole-field interactions between periodic images. Density of states (DOS) calculations were performed with a finer k-mesh of 11x11x1 within a non-self-consistent calculation, using the density obtained from a self-consistent calculation with a 7x7x1 k-mesh.

The initial M1 and M9 structures used in the calculations were obtained from the work of Mete and coworkers (*63*). We utilized their coordinates to carry out our own calculations of the molecule in the gas-phase with the full thiol moiety. For condensed phase modeling, the carboranethiol was adsorbed at the hexagonal-closed-packed (hcp) and face-centered-cubic (fcc) hollow sites on a 3x3, five-layer thick Au (111) periodic slab supercell, without any H atom bound to S. The 3x3 cell was constructed based on integer scaling of the lateral primitive unit cell for Au with a fixed DFT-PBE-D3-predicted equilibrium bulk lattice constant of 4.10 Å, which compares favorably with the experimental value of 4.08 Å (*64*). A vacuum layer of 20 Å thickness was used to keep periodic images of the surfaces from interacting. The structures used for the adsorbates on Au assume the H atoms attached to S first dissociatively adsorbed and then desorbed, given the weak binding of H to Au. Structures adsorbed at the fcc site were found to be lowest in energy. The S-Au bond lengths have average values of 2.444 Å and 2.438 Å for the M1 and M9 structures, respectively. The M9 structure is lower in energy by -0.81 eV compared to M1, in agreement with relative energy trends reported in DFT-PBE calculations of the molecules adsorbed on Au at a lower concentration (*63*).

Calculations also were performed using embedded correlated wavefunction (ECW) theory within the density functional embedded theory (DFET) formalism (*38*). In DFET, the embedding potential is optimized using DFT to account for the interaction between an embedded region (here a metal cluster) and the extended surroundings (here a metal surface), and then the embedded region is subsequently treated at higher levels of theory using CW methods in the presence of the DFET-derived embedding potential. The embedding potential is included as an additional term in the electronic Hamiltonian

$$(H_{el} + V_{emb})\psi_{cl} = E_{cl,emb}\psi_{cl} \tag{S3}$$

In the above equation, H_{el} is the electronic Hamiltonian, V_{emb} is the embedding potential, ψ_{cl} is the wavefunction describing the embedded cluster, and $E_{cl,emb}$ is the energy of the embedded cluster. The ECW energy, E_{ECW}, corrects the DFT description of the total system, E_{sys}^{DFT}, by replacing the DFT description of the embedded cluster, $E_{cl,emb}^{DFT}$, with a CW description, $E_{cl,emb}^{CW}$

$$E_{ECW} = E_{sys}^{DFT} - E_{cl,emb}^{DFT} + E_{cl,emb}^{CW}. \quad \text{(S4)}$$

The embedding potential was optimized at the DFT-PBE level for a seven-atom Au cluster within a bare 5x5, five-layer-thick Au (111) periodic slab, using an in-house embedding potential optimization code (*47*) that runs in conjunction with VASP. The embedding potential optimization used the same PW basis kinetic energy cutoff of 600 eV and the same Methfessel-Paxton Fermi surface smearing (width of 0.09 eV) as the original DFT calculations above. Brillouin zone sampling via the Monkhorst-Pack method used Γ-point-centered k-meshes of 1x1x1 and 5x5x1 for the cluster and environment, respectively. The structures for the M1 and M9 carboranethiol molecules obtained from the 3x3 unit cell periodic DFT calculations mentioned earlier were translated onto the fcc hollow site on a seven-atom Au cluster (five atoms in the top layer, two in the second) excised from the optimized, bare 5x5 Au(111) slab. The structures are available as .cif files. The same embedding potential – the interaction potential between the seven-atom Au cluster and the remaining atoms in the 5x5 five-layer slab surrounding it was used for both M1 and M9 molecules adsorbed on the seven-atom Au cluster.

The ECW calculations were performed using the complete active space self-consistent field (CASSCF) method (*37*), with several active spaces tested. The CASSCF active space used here (10 electrons in 10 orbitals) only consists of Au electrons and orbitals, with no electron correlation included on the carboranethiol molecule because the dominant correlation effects are in fact in the metal. Thus, the carboranethiol molecule effectively is treated at the Hartree-Fock (HF) (*42*) level within the CASSCF calculation, so a consistent treatment of the molecule requires that its gas phase analog be calculated with HF theory. Thus, HF calculations were performed to obtain the dipole moments of the individual carboranethiol molecules in the gas-phase (H-capped, *i.e.*, the parent thiols). We then could approximately decompose the contributions of the adsorbed molecule and the Au surface from the dipole moment of the total system. This treatment maintains consistency of the theoretical treatment of the molecule while including static correlation needed to describe the Au electrons properly because of the near degeneracies of metal states near the Fermi level. Molpro (*48, 59*) version 2012.1 was utilized for embedded CASSCF and gas-phase HF calculations. The embedding integral matrices to include in the Hamiltonian of the embedded cluster calculations were represented in the contracted (atomic orbital) Gaussian basis, using our open-source, standalone code available on the Carter group github site (*38*).

ECW calculations are performed within Gaussian-type-orbital (GTO) basis sets. We used Dunning's cc-pVDZ basis set for H (*65*) and the Stuttgart relativistic large core effective core potentials (ECPs) (*66*) for C, B, and S, in conjunction with the corresponding Stuttgart basis sets provided on the EMSL (6*7, 68*) basis set exchange database. The ECPs utilized for these elements were the ECP2MWB and ECP10MWB Stuttgart ECPs (*66*), which implicitly treat the nuclei and the two core [1*s*] electrons for C and B and the 10 core [1*s*2*s*2*p*] electrons for S, using multi-electron quasi-relativistic fitting. The ECP utilized for Au was the ECP60MDF (*69*) Stuttgart ECP, which subsumes 60 core electrons and the nucleus into the ECP with multi-electron fully relativistic fitting, in conjunction with Peterson's and Puzzarini's VDZ-PP basis set (*74*).

Gas-phase-optimized carboranethiol structures were obtained from GTO-DFT-PBE calculations using the ECPs and basis sets described above. Their dipole moments were calculated to verify the accuracy of these GTO basis sets, benchmarked against gas-phase calculations with a larger def2-TZVP (*75*) basis set, and also against dipole moments calculated with the more accurate B3LYP (*72*, *73*) functional as reported in the literature (*27*). The resulting dipole moments were sufficiently close in magnitude and orientation to suggest a similar description of the electronic structure, lending

credibility to the use of these basis sets and ECPs for the current application. Total dipole moments then were calculated for the M1 and M9 molecules adsorbed on the embedded cluster using CASSCF theory as described above. The adsorbate structures used for the embedded CASSCF calculations then were capped with H atoms and kept frozen, only optimizing the thiol H in a PW-DFT-PBE calculation. The parameters utilized for the PW calculations were the same as before, evaluated at the Γ-point, with a supercell size equivalent to the 5x5 slab. The H-cap-relaxed frozen M1 and M9 structures then were utilized in HF calculations to obtain an estimate of the molecular dipoles. Dipole differences then were calculated from the total dipole moment of the embedded cluster with adsorbed M1 or M9 followed by subtraction of the dipole moments of the H-capped, frozen gas-phase molecules to estimate the induced dipole at the Au surface.

Section S5. Dipole moments of carboranethiols and gold

The dipole moments calculated for the carboranethiol molecules adsorbed on Au ("Total") and in the gas-phase thiols ("Gas-Phase Thiol"), and the resulting induced dipole moments of the Au surface ("Au Surface Induced") are listed in Table S1. It is important to clarify that the theory employed here, omitting the solvent ethanol, was not used to assess energetics; it was only used to examine electronic structure (i.e., dipoles and densities of states) to help interpret the experiments. We do not expect the electronic structure of Au or M1 or M9 to be affected by solvation because of the random orientations of the ethanol molecules due to their expected weak interaction with Au and M1 and M9. To properly model any potential partial ordering of ethanol molecules around M1 and M9 would require an extensive set of ab initio molecular dynamics simulations, which is beyond the scope of the present work.

Table S1. Calculated dipole moment vector components in debye (D) for M1 and M9 carboranethiol molecules adsorbed on Au ("Total") and in the gas-phase ("Gas-Phase Thiol"). "Au Surface Induced" is the difference between the two dipole moments, which is used to estimate the Au surface induced dipole moment. The dipole magnitudes, $|\vec{\mu}|$, are also presented. We calculated the total dipole moments with embedded CASSCF using a (10e,10o) active space. We employ the HF method for the gas-phase molecules with the same basis sets as the CASSCF calculations. The cc-pVDZ (*65*) basis set was used for H. The Stuttgart ECP2MWB ECP (*66*) was used for C and B atoms, and the ECP10MWB ECP (*66*) was used for S atoms in conjunction with the corresponding Stuttgart basis sets available through the EMSL database (*67*, *68*). The Stuttgart ECP60MDF ECP (*69*) and VDZ-PP (*70*) basis set were used for Au.

Dipole moment components	X	Y	Z	$\lvert\vec{\mu}\rvert$
M1				
Total	1.07	1.44	0.29	1.81
Gas-Phase Thiol	1.20	-2.59	-1.18	3.09
Au Surface Induced	-0.13	4.03	1.47	4.29
M9				
Total	-0.03	4.84	7.29	8.75
Gas-Phase Thiol	0.23	2.10	3.75	4.30
Au Surface Induced	-0.25	2.73	3.53	4.47

The results of the calculations presented in Table S1 and shown in Fig. 3 in the main text reveal that the M1 and M9 systems exhibit very different surface interactions. The induced dipole moment for the Au surface in the M1 system compensates for the x and y components (i.e., in the surface plane) of the dipole in the M1 adsorbate and orients anti-parallel to the dipole of the M1 adsorbate. On the other hand, the dipole for the Au surface in the M9 system aligns head-to-tail with the z component of the dipole in the M9 adsorbate. It is the in-plane component (x and y components) of the induced dipole that will have the greatest damping effect on the plasmon. The potential energy of two interacting dipoles is (*74*)

$$E(r) = -\frac{1}{4\pi\epsilon_0|\vec{r}_{12}|^3}\left(\frac{3(\vec{\mu}_1\cdot\vec{r}_{12})(\vec{\mu}_2\cdot\vec{r}_{12})}{|\vec{r}_{12}|^2} - (\vec{\mu}_1\cdot\vec{\mu}_2)\right) \quad \text{(S5)}$$

In Eq. S5, $\vec{\mu}_1$ and $\vec{\mu}_2$ are the two interacting dipole moment vectors. The energy from the interaction, $E(r)$, requires the dot product of the two dipole moments and a dot product of the dipoles with the vector connecting their centers, $\vec{r}_{12}$. The M1/Au system has a larger Au surface induced dipole compared to M9/Au within the x-y plane (Table S1). The magnitudes of the in-plane dipoles are 4.03 and 2.74 D (or 1.57 and 1.07 ea_0) for M1 and M9, respectively. Including subtraction of the embedded bare Au cluster dipole moment contribution changes the M1:M9 induced in-plane dipole moment ratio only slightly, to 1.45:1 instead of the 1.47:1 reported in the main text. This negligible change is expected because the embedded bare Au cluster should have a negligible moment (predicted to have x (y)-components of 0.15 (-0.09) D). Subtracting these values from the moment components below gives another estimate for the magnitudes of the in-plane induced dipole moments for M1 and M9 of 4.13 and 2.85 D, respectively, compared to 4.03 and 2.74 D reported above and in the main text.

Note also that due to the nature of the orientations of the dipoles for M1 and M9, the M1 dipoles will align in the x and y directions head-to-tail when there is complete surface coverage, enlarging the dipole magnitude for M1 adsorbates slightly. The actual M9/Au saturated coverage on the surface will align the dipoles head-to-head and tail-to-tail, resulting in a further decrease of the local magnitude of these dipole moments per adsorbate.

Halas and coworkers previously found that the magnitudes of dipole moments of adsorbed molecules were inversely related to the decay lifetimes of excited electrons in Au (*52*). Within that work, theoretical calculations were performed to obtain the dipoles of adsorbates on copper surfaces to approximate the adsorbate-Au interactions, since it was too computationally demanding at the time to study Au surfaces directly. For aniline, they determined that the parallel surface orientation, which would exhibit a dipole parallel to the surface, agreed with experimental trends whereas aniline oriented perpendicularly did not. Following this work, we suggest that the magnitude of the dipole moment's parallel component is correlated with the longitudinal plasmon excitation decay lifetime in nanorods, which is inversely proportional to the amount of spectral broadening observed. The ratio between the induced in-plane dipoles by M1 and M9 is 1.47:1, which agrees well with the experimental ratio for the equilibrium plasmon linewidth broadening of 1.56:1.

Section S6. Density of states and Persson theory

Persson *et al.* derived the change in the plasmon linewidth, due to damping from a resonance state on the adsorbate, using a jellium model and approximating the shape of the nanoparticle as a sphere (*17, 76*). The change in plasmon linewidth due to chemical interface damping correlates directly with the cross-section for diffuse scattering

$$\Delta\Gamma \approx \frac{3v_F n_a \vartheta_{diff}(\omega)}{8R} \quad \text{(S6)}$$

$$\vartheta_{diff}(\omega) \propto \int_{\epsilon_F-\hbar\omega}^{\epsilon_F} d\epsilon[\epsilon\Gamma(\epsilon)\rho_a(\epsilon+\hbar\omega) + (\epsilon+\hbar\omega)\Gamma(\epsilon+\hbar\omega)\rho_a(\epsilon)] \quad \text{(S7)}$$

In Eq. S6, ΔΓ is the contribution to the surface plasmon width, v_F is the Fermi velocity of the electron, n_a is the number of adsorbed molecules, $\vartheta_{diff}(\omega)$ is the diffuse scattering cross-section, and R is the sphere radius that can be replaced with an effective path length (*23*) for the nanorods examined here. $\vartheta_{diff}\ (\omega)$ can be approximated in Eq. S7 by the integral of the product of the adsorbate DOS, $p_a(\epsilon)$, the excitation linewidth, $\Gamma(\epsilon)$, and the energy, ϵ. The integral is evaluated for energies with respect to vacuum, suggesting that ϵ_F is the workfunction of the metal surface.

The first term in Eq. S7 accounts for the overlap between available hot electrons from the occupied space of the metal with the virtual space of the adsorbate DOS. The second term in Eq. S7 describes the transfer of hot holes to the adsorbate. The complete DOS for each atom in the system is provided in fig. S7 and S8 for M1 and M9, respectively. A close-up view of the projected DOS for Au and S atoms for the M1 and M9 systems are presented in fig. S9a and S9b. Both the S-projected DOS and Au-projected DOS for M1 and M9 match very closely with little comparative difference, indicating little difference in the availability of hot electrons and hot holes in both Au and S. Because the band gaps for both systems are quite large (fig. S7 and S8), we evaluate Persson's equation by testing for the contributions from resonance states located on S atoms. The two Lorentzians are centered near the Fermi edge $(\epsilon_F + \Gamma(\epsilon)/2)$ for hot electrons and near the excitation wavelength below the Fermi level $(-\hbar\omega + \epsilon_F + \Gamma(\epsilon)/2)$ for hot holes. The corresponding values for the excitation energy ($\hbar\omega$), the work function for Au (*35*) to define the Fermi level, ϵ_F, and the plasmon linewidth, $\Gamma(\epsilon)$ are 1.72 eV, 5.3 eV, and 0.09 eV, respectively. Evaluating both terms in Eq. S7 in the Lorentzian-projected S-atom DOS provides a ratio of 1.01:1 for M1 over M9 for the integrated portion of the diffuse scattering cross-sections. fig. S9c illustrates the hot-electron and hot-hole functions expressed in the integrand in Eq. S7. Thus, interaction with near-Fermi resonance states is not the predominating mechanism due to equivalent contributions to ΔΓ from both M1 and M9.

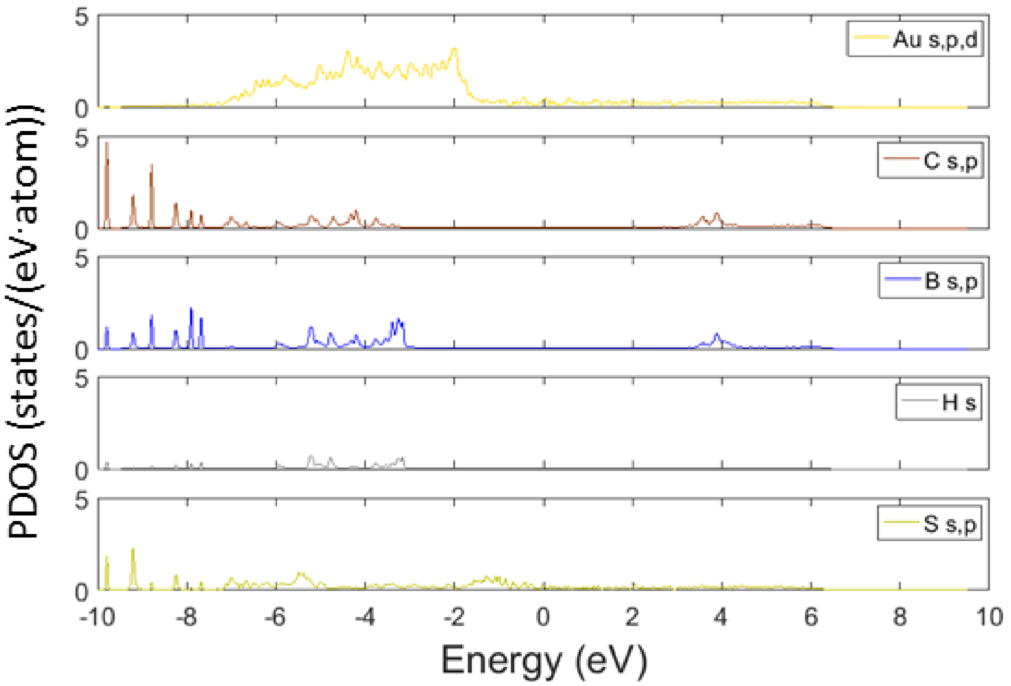

Fig. S7. The projected DOS for the atoms comprising the M1 carboranethiol molecule adsorbed on Au in a face-centered cubic (fcc) hollow site, according to DFT-PBE-D3 calculations. The DOS is decomposed into the contributions from each atom in the system.

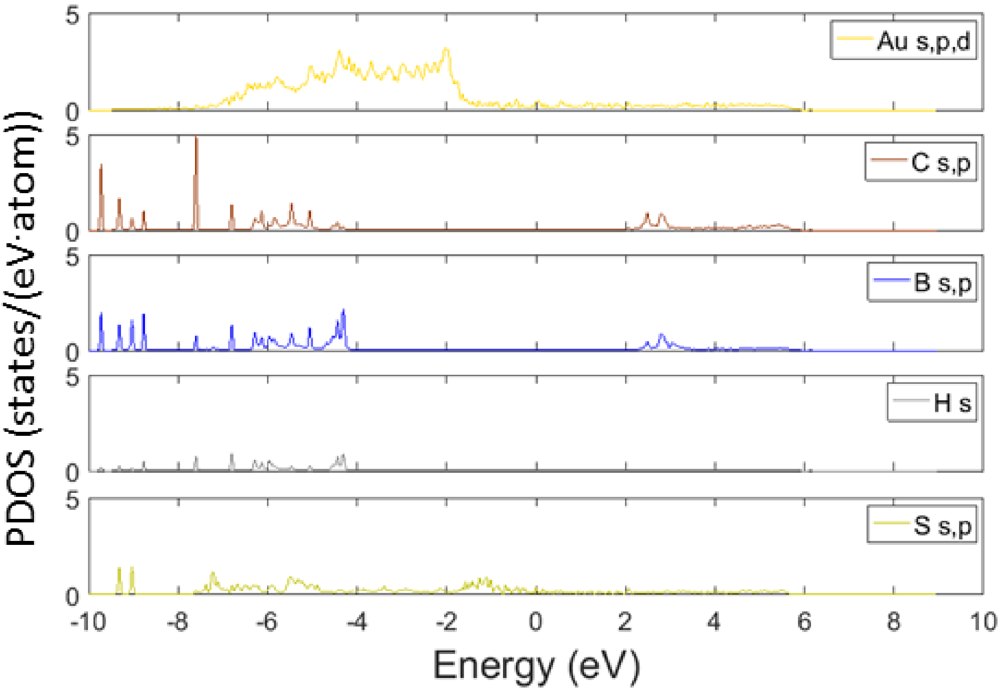

Fig. S8. The projected DOS for the atoms comprising the M9 carboranethiol molecule adsorbed on Au in an fcc hollow site, according to DFT-PBE-D3. The DOS is decomposed into the contributions from each atom in the system.

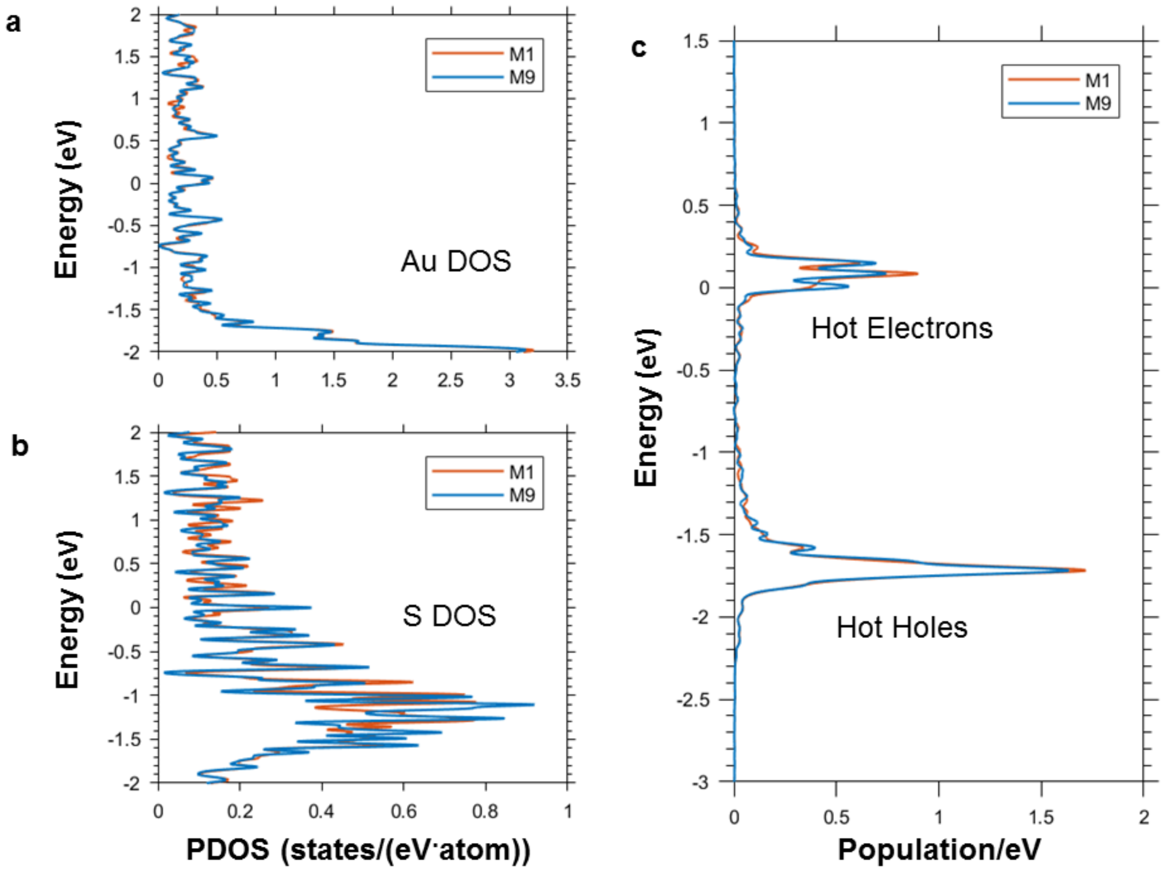

Fig. S9. Predicted projected DOS and Lorentzian functions for M1 and M9 molecules on Au(111). a) Au-projected DOS and b) S-projected DOS for M1 (orange) and M9 (blue) adsorbates on an Au periodic slab according to DFT-PBE calculations. c) Projected Lorentzian functions (integrand from Eq. S7) for hot holes and hot electrons (orange – M1) (blue – M9). The Fermi level for Au was assigned a value of 5.3 eV for the calculations but is given a relative value of 0 eV in all figures.
